# Herbal medicine (Zhengan Xifeng Decoction) for essential hypertension protocol for a systematic review and meta-analysis

**DOI:** 10.1097/MD.0000000000014292

**Published:** 2019-02-08

**Authors:** Yonglian Huang, Yanhong Chen, Hairong Cai, Dongjie Chen, Xiaoming He, Zhixiong Li, Xiaojing Cai, Xiaohong Peng, Yaxiu Huang, Shaoping Li, Qifeng Cao, Ping Wang, Bojun Chen

**Affiliations:** a Department of Critical Care Medicine, Beijing University of Chinese Medicine Shenzhen Hospital; b The Second Clinical College, Guangzhou University of Chinese Medicine; c Department of Emergency, Foshan Hospital of Traditional Chinese Medicine; d Department of Emergency, The Second Affiliated Hospital of Guangzhou University of Chinese Medicine, Guangzhou, Guangdong Province, China.

**Keywords:** essential hypertension, protocol, systematic review, Zhengan Xifeng Decoction

## Abstract

**Background:**

Essential hypertension is one of the most common chronic diseases worldwide, as well as a leading risk factor for cardiocerebrovascular diseases. Zhengan Xifeng Decoction (ZGXFD) has been widely used to treat essential hypertension, but there is no systematic review by assessing efficacy and safety of ZGXFD on essential hypertension. Therefore, we aim to perform systematic review and meta-analysis to evaluate the efficacy and safety of ZGXFD in the treatment of essential hypertension.

**Methods:**

This systematic review and meta-analysis will be performed by means of electronic databases, including EMBASE, Cochrane Center Registration Controlled trials (Cochrane Library), Web of Science (WOS), World Health Organization International Clinical Trials Registry Platform, PubMed, China Biomedical Literature Database (CBM), China National Knowledge Infrastructure (CNKI), Chinese Scientific Journal Database (VIP), and Wan-fang database. The electronic databases will be searched from their inception to October 2018. This systemic review will include only published English and Chinese articles randomized controlled trials (RTCs) of ZGXFD on essential hypertension. The primary outcome is Efficacy and blood pressure (BP), blood lipid and adverse reactions will be accepted as secondary outcomes. All statistical analyses will be conducted using RevMan V.5.3.5 software.

**Results:**

This systematic review and meta-analysis will provide high-quality evidence from several aspects, including for efficacy, blood pressure, blood lipid and adverse effects to evaluate the efficacy and safety of ZGXFD on EHTN.

**Conclusion:**

This systematic review will determine whether or not ZGXFD is an effective intervention for essential hypertension.

## Abstract 2

Supplemental Digital Content is available in the text

## 1 Introduction

Essential hypertension is the most common chronic disease and leading risk factor for cardiocerebrovascular diseases. Stroke, myocardial infarction, heart failure, and chronic kidney disease increase morbidity and mortality. Essential hypertension consuming health care and social resources severely have placed a heavy burden on families and countries and become an important public health problem in China.^[1–3]^ With the changes of people's lifestyle and the acceleration of population aging process, the prevalence of hypertension in China has increased from 5.11% in 1959 to 17.65% in 2002.^[4]^ The number of patients with essential hypertension has exceeded 300 million currently in China,^[5]^ the prevalence of hypertension was 29.6%, and the prevalence of men was higher than that of women (31.2% and 28.0%, respectively). The awareness, management, and control rate of all hypertensive patients were 42.6%, 34.1%, 9.3%, and 27.4%, respectively.^[6–8]^ Chronic diseases have become the leading threat to health, which accounts for 80% of all causes of death in China. Cardiocerebrovascular diseases are the leading cause of chronic disease death; 50% to 75% of strokes and 40% to 50% of myocardial infarctions are associated with increased hypertension.^[9]^ The medical care of patients with hypertension costs >36.6 billion annually.^[10,7]^ Hypertension is a disease that can be prevented and controlled. The reduction in blood pressure could reduce stroke and heart disease events, improve the quality of life of patients, and reduce the burden of disease significantly. Antihypertensive agents are the mainstay of the treatment, including diuretics, beta-adrenergic blocking agents, angiotensin-converting enzyme inhibitor (ACEI), angiotensin II receptor inhibitor (ARB), and calcium channel blocker (CCB). However, due to costs, adverse effects, complications, and so on, near half of the patients are unable to control blood pressure through drug therapy effectively.^[11]^ Life behavior interventions such as exercise, weight loss, and salt intake control can also be used to lower blood pressure, but difficult to adhere to take.

Traditional Chinese Medicine (TCM) is an important part of complementary and alternative medicine (CAM). Although the mechanism is not clear, it has been widely accepted in China and applied in clinical practice.^[12]^ A large number of research have shown that Chinese medicine, acupuncture and taichi, could treat hypertension effectively.^[13–16]^ Zhengan Xifeng Decoction (ZGXFD) is made up of 12 kinds of traditional Chinese medicines: Nxiuxi (*Achyranthes bidentata* Blume), Daizheshi (Haematite), Longgu (Fossilia OssiaMastodi), Muli (Ostrea gigas thumb), Guiban (Carapax testudinis), Baishao (*Paeonia lactiflora* pall), Xuanshen (*Scrophularia ningpoensis* hemsl), Tiandong (*Asparagus cochinchinensis* (Lour.) Merr.), Chuanlianzi (*Melia toosendan*), Maiya (Fructus hordei germinatus), Yinchen (*Artemisia capillaris* thunb), Gancao (Glycyrrhizae radix et rhizoma). Animal experiments have shown that ZGXFD could lower blood pressure, inhibit the expression of angiotensin II, endothelin, secretin, and somatostatin in rats with essential hypertension,^[17,18]^ and the apoptosis of vascular smooth muscle.^[19]^ Clinical researches have also shown that there is good curative effect for ZGXFD in the treatment of essential hypertension ^[20,21]^; however, there is a lack of systematic review and meta-analysis regarding its efficacy and safety. Therefore, we developed the protocol for a systematic review and meta-analysis to assess the efficacy and safety of ZGXFD in the treatment of essential hypertension, which may provide a reference for clinical application.

## 2 Methods

### 2.1 Inclusion criteria for study selection

#### 2.1.1 Types of studies

Only randomized controlled trials (RCTs) of ZGXF in the treatment of essential hypertension will be eligible for inclusion, whether blinding is used or not. Cohort studies, review articles, controlled (nonrandomized) clinical trials (CCTs), Letters to editor, comments will be excluded. There is no limitation in language and time.

#### 2.1.2 Types of patients

Patients (18 years of age and older) with essential hypertension, diagnosed by hospital outpatient or inpatient, will be included. The diagnostic criteria for hypertension will be developed according to the New ACC/AHA Hypertension Guidelines for the prevention, detection, evaluation, and management of high blood pressure in adult^[22]^ and 2010 Chinese guidelines for the management of hypertension^[23]^: systolic blood pressure (SBP) ≥140 mm Hg and/or diastolic blood pressure (DBP) ≥90 mm Hg (1 mm Hg = 0.133 kPa), or antistress drugs are being used. The patient's age, sex, race, nationality, and comorbidity are not limited. We will exclude animal studies and trials that are primarily conducted in children (17 years of age and younger).

#### 2.1.3 Types of interventions

Patients in the experimental group have been treated with ZGXFD, and the control group treated with a placebo or blank control group or recommended antihypertensive agents (including diuretics, beta-adrenergic blocking agents, CCB, ACEI, ARB, and so on). If interventions of the experimental group were ZGXFD combined with antihypertensive drugs, the same antihypertensive drugs must be used in the control group. The administration time of each group is not <4 weeks.

#### 2.1.4 Types of outcome measures

##### 2.1.4.1 Primary outcomes

The primary outcomes are determined according to the 2010 Chinese guidelines for the management of hypertension^[22]^: markedly effective—diastolic blood pressure drop >20 mm Hg or decreased <10 mm Hg (1 mm Hg = 0.133 kPa), but has reached the normal range of blood pressure; effective—diastolic blood pressure decreased >10 to 19 mm Hg or decreased <10 mm Hg, but has not reached the normal range the normal range of blood pressure; invalid—did not meet the above criteria.

##### 2.1.4.2 Secondary outcomes

The secondary outcome includes SBP, DBP, total cholesterol (TC), triglycerides (TG), high-density lipoprotein (HDL), low-density lipoprotein (LDL), body mass index, and adverse reactions (nausea, vomiting, diarrhea, and so on).

### 2.2 Search methods for the identification of studies

Nine electronic databases including EMBASE, Cochrane Library, WOS, World Health Organization International Clinical Trials Registry Platform, PubMed, CBM, CNKI, VIP and Wan-fang database will be searched their inception to October 2018 for the relevant RCTs of ZGXFD for essential hypertension. Search terms to be used will include essential hypertension, ZGXFD, and RCTs. The strategy for searching the PubMed will be shown as an example in Appendix A (Supplemental Appendix A), and modified by using other databases.

#### 2.2.1 Searching other resources

In the meantime, we will manually search for references retrieved literature to identify any relevant gray literature and search Google Scholar to avoid missing relevant studies on the Internet. Furthermore, we will contact experts to see if they understand other research topics.

### 2.3 Data collection and analysis

#### 2.3.1 Selection of studies

**Figure 1:**
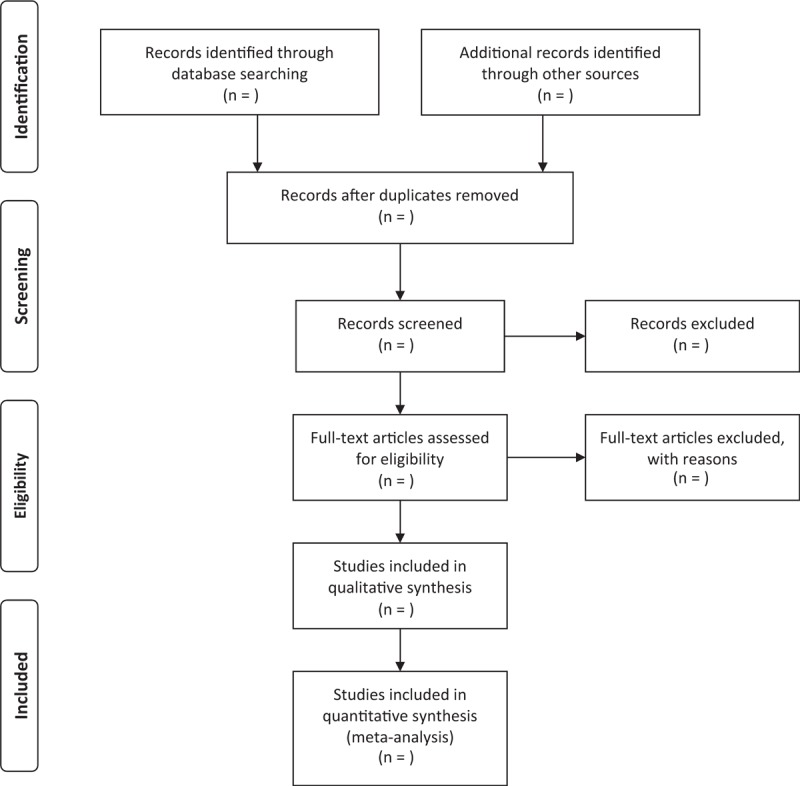
Preferred Reporting Items for Systematic review and Meta-Analysis (PRISMA) flow chart.

The study selection will be conducted by 2 authors (HRC and YHC) according to the above inclusion criteria independently. The results will be exported to the endnote referencing software (version 9.0, Thomas Reuters, CA) and duplicate studies will be excluded. Initially, the independent authors will read the titles and abstracts to exclude articles that are not in compliance with the inclusion criteria clearly. In the second process of selection, the full-text of the articles will be reviewed and assessed to determine whether it truly meets the inclusion criteria or not. The results will be double checked for accuracy finally. When there are any disagreements, the matter will be resolved by discussion or consulting with a third author. The process of study selection and meta-analysis is presented in a in an adapted PRISMA flow diagram (Figure 1).

#### 2.3.2 Data extraction and management

The information will be extracted by a standardized extraction form, which is carried out independently by 2 authors. The data to be extracted will include general information of the research, research methods and possible biases, characteristics of the research objects, intervention measures, outcome indicators, results, and other information that needs to be collected. The results were checked by the 2 independent authors. In cases where agreement cannot be reached, the matter will be resolved with a third author.

#### 2.3.3 Assessment of risk of bias in included studies

The methodological quality of the included literature will be evaluated by using “risk of bias” tool for RCTs recommended by the Cochrane Handbook V.5.1. Quality will be evaluated using the following: random sequence generation, random allocation concealment, blind method implementation, outcome assessor, result data integrity, selective outcome report, and other biases. The methodology for each item was graded as a high risk of bias, low risk of bias, and uncertain bias in accordance with the quality classification criteria. The risk bias assessment is independently conducted by the 2 authors. When there is a disagreement, it will be resolved by discussion or consulting with a third author.

#### 2.3.4 Measures of treatment effect

The relative risks (RR) with 95% confidence intervals (CIs) will be presented for dichotomous data, and standardized mean difference (SMD) with 95% CIs for continuous outcomes.

#### 2.3.5 Dealing with missing data

If information in the research reports is incomplete, the authors will contact the corresponding author or the first author by phone or email to obtain the necessary data. If no additional information is available, data synthesis will be performed based on the available data. But at the same time, we will also address the potential impact of missing data. If necessary, the articles will be excluded.

#### 2.3.6 Assessment of heterogeneity

The heterogeneity will be assessed by χ^2^ test (*a* = 0.1) and *I*^2^ statistic. The heterogeneity will be considered small if *I*^2^ < 25%, *I*^2^ > 25% and < 50% moderate heterogeneity, and *I*^2^ > 50% large heterogeneity. If *I*^2^ > 50%, a subgroup analysis will be performed to investigate the underlying cause of clinical or method heterogeneity.

#### 2.3.7 Assessment of reporting bias

If >10 studies are included, we will analyze the impact of publication bias based on the funnel plot of Revman Manager software (version 5.3; The Cochrane Collaboration, Oxford, UK).

#### 2.3.8 Data synthesis

Data synthesis will be performed by using RevMan software V.5.3 provided by the Cochrane Collaboration. When *P* > .1 and *I*^2^ < 50%, a fixed-effects model will be conducted for meta-analysis, whereas when *P* < .1 and *I*^2^ ≥50%, heterogeneity analysis will be performed to find the source of heterogeneity. Then subgroup analysis will be conducted according to the source of heterogeneity. In the subgroup analysis, if there is statistical heterogeneity between the studies but no clinical heterogeneity, a random effects model will be used for meta-analysis. Descriptive analysis will be performed if the heterogeneity is too large or the source of heterogeneity is unknown.

#### 2.3.9 Subgroup analysis

Subgroup analysis is to explore the source of heterogeneity. Subgroup analysis will be performed according to different interventions, participants, sex, duration of disease, and dose of medication, if >10 studies are included.

#### 2.3.10 Sensitivity analysis

We will perform sensitivity analysis based on sample size, missing data results, and methodological quality.

#### 2.3.11 Grading the quality of evidence

The quality level of evidence will be analyzed by using GRADE profiler software (Version 3.6, The GRADE Working Group).

## 3 Discussion

Hypertension is the most common chronic disease in the clinic and an important risk factor for cardiocerebrovascular diseases. Antihypertensive agents are the main method for treating hypertension, but clinical applications are limited due to treatment costs, adverse effects, and complications. Life behavior interventions such as exercise, weight loss, and salt intake control can also be used to lower blood pressure, but these interventions are difficult to adhere to. Studies have shown that ZGXFD could improve clinical symptoms such as dizziness and headache and lower blood pressure. However, there is no systematic review and meta-analysis regarding its effects and safety. Therefore, a high-quality systematic review and meta-analysis is necessary. We hope that this systematic review will provide more convincing evidence for ZGXFD in the treatment of essential hypertension. However, there may be some potential shortcomings in this systematic review. First, heterogeneity risk will engender due to different doses, age, and severity of hypertension. Second, small samples may lead to high risks of bias.

## Author contributions

**Conceptualization:** Xiaoming He.

**Data curation:** Xiaohong Peng, Yaxiu Huang.

**Funding acquisition:** Bojun Chen.

**Investigation:** Shaoping Li, Qifeng Cao.

**Methodology:** Zhixiong Li, Dongjie Chen.

**Project administration:** Ping Wang, Bojun Chen.

**Software:** Xiaojing Cai.

**Supervision:** Ping Wang, Bojun Chen.

**Validation:** Dongjie Chen.

**Writing – original draft:** Yonglian Huang, Yanhong Chen, Hairong Cai.

**Writing – review and editing:** Yonglian Huang, Yanhong Chen, Hairong Cai.

## Supplementary Material

Supplemental Digital Content
