# Supplementary material for: Herbal medicine (Zhengan Xifeng Decoction) for essential hypertension protocol for a systematic review and meta-analysis
Source: Medicine (Baltimore). 2019 Feb 8;98(6):e14292. doi: 10.1097/MD.0000000000014292 (PMC6380723; doi:10.1097/MD.0000000000014292)
Supplement: Supplemental Digital Content [file medi-98-e14292-s001.docx]

**Appendix A.**

***Search strategy used in PubMed database***

#1 essential hypertension OR hypertension OR high blood pressure OR high blood pressures

#2 zhengan xifeng decoction OR zhen gan xi feng decoction OR zhengan xifeng tang OR zhengan xifeng yin

#3 Randomized controlled trial OR clinical study OR Clin-ical Trial OR Controlled study OR Controlled Trial OR Random*Control* study OR random* Control* Trial

#1 AND #2 AND #3
